# A common *NTRK2* variant is associated with emotional arousal and brain white-matter integrity in healthy young subjects

**DOI:** 10.1038/tp.2016.20

**Published:** 2016-03-15

**Authors:** K Spalek, D Coynel, V Freytag, F Hartmann, A Heck, A Milnik, D de Quervain, A Papassotiropoulos

**Affiliations:** 1 Division of Cognitive Neuroscience, Department of Psychology, University of Basel, Basel, Switzerland; 2 Division of Molecular Neuroscience, Department of Psychology, University of Basel, Basel, Switzerland; 3 Psychiatric University Clinics, University of Basel, Basel, Switzerland; 4 Department Biozentrum, Life Sciences Training Facility, University of Basel, Basel, Switzerland

## Abstract

Dysregulation of emotional arousal is observed in many psychiatric diseases such as schizophrenia, mood and anxiety disorders. The neurotrophic tyrosine kinase receptor type 2 gene (*NTRK2*) has been associated with these disorders. Here we investigated the relation between genetic variability of *NTRK2* and emotional arousal in healthy young subjects in two independent samples (*n*_1_=1171; *n*_2_=707). In addition, diffusion tensor imaging (DTI) data in a subgroup of 342 participants were used to identify *NTRK2*-related white-matter structure differences. After correction for multiple testing, we identified a *NTRK2* single nucleotide polymorphism associated with emotional arousal in both samples (*n*_1_: *P*_nominal_=0.0003, *P*_corrected_=0.048; *n*_2_: *P*_nominal_=0.0141, *P*_corrected_=0.036). DTI revealed significant, whole-brain corrected correlations between emotional arousal and brain white-matter mean diffusivity (MD), as well as significant, whole-brain corrected *NTRK2* genotype-related differences in MD (*P*_FWE_<0.05). Our study demonstrates that genetic variability of *NTRK2*, a susceptibility gene for psychiatric disorders, is related to emotional arousal and—independently—to brain white-matter properties in healthy individuals.

## Introduction

A dysregulation of emotional processes is commonly observed in many psychiatric disorders. Specifically, more than 75% of psychiatric diagnostic categories are characterized by emotional dysregulation.^1^ In some psychiatric disorders, like mood or anxiety disorders, emotional dysregulation is one of the core symptoms giving rise to the respective diagnosis.^1^

The International Affective Picture System (IAPS),^2^ which consists of a large set of standardized, emotion-evoking photographs covering a wide range of semantic categories, is often used in experimental studies to test and quantify emotional processes. Typically, subjects are asked to rate these pictures on two emotion dimensions, namely valence (ranging from pleasant to unpleasant) and arousal (ranging from calm to excited).^2^ Differences in ratings of IAPS pictures have been observed in patients with psychiatric disorders.^3^ Several studies demonstrated altered arousal ratings of IAPS pictures in schizophrenia, borderline personality disorder, major depressive disorder and substance abuse.^4, 5, 6, 7, 8, 9^

Heritability of psychiatric disorders is generally high with heritability estimates ranging between 40 and 80%,^10, 11, 12, 13, 14, 15^ suggesting substantial genetic contribution to disease risk. The gene encoding the neurotrophic tyrosine kinase receptor type 2 (*NTRK2* also known as *TRKB*) has been often discussed in this context, mostly due to its involvement in the neurotrophic hypothesis of stress-related psychiatric disorders.^16, 17^ NTRK2 can be activated by several neurotrophins including brain-derived neurotrophic factor (BDNF), neurotrophin 3 (NT3) and NT4/5.^18, 19, 20, 21^ TrkB signaling triggered by neurotrophins plays a central role in cell proliferation, survival and differentiation, as well as synaptic plasticity, and neurotransmitter release.^22, 23, 24, 25, 26^ Specifically, NTRK2, which activates several intracellular signaling cascades on ligand binding, is known to interact with expression and trafficking of glutamate receptors.^26, 27^ Importantly, NTRK2 modulates specific phases of fear learning and synaptic plasticity in the amygdala,^28^ a brain region centrally involved in the processing of emotionally arousing stimuli and in the pathogenesis of psychiatric conditions.^29, 30^ Several recent studies have indeed suggested a relationship between *NTRK2* and a broad range of psychiatric disorders such as depression, schizophrenia, addiction, eating and anxiety disorders.^31, 32, 33, 34, 35, 36, 37, 38, 39, 40^ For example, Ernst *et al.*^33^ identified a deletion in a human *NTRK2* promoter and provided evidence for an involvement of this deletion in anxiety traits. In two independent samples of depressed patients, Kohli *et al.*^37^ observed an association between lifetime history of suicide attempts and a combination of several independent risk alleles within the *NTRK2* gene. In addition, a study also points to an involvement of genetic *NTRK2* variants in brain imaging parameters, such as genotype-dependent differences in white-matter properties in depressed patients.^41^ In support of the genetic data, the expression pattern of NTRK2 in the brain, with high expression levels in different brain regions such as the occipital, temporal and frontal cerebral cortices, the putamen and the cerebellar cortex,^18, 42, 43^ also argues against a role for NTRK2 in a specific psychiatric disorder. The available data rather point to an involvement of NTRK2 in general mental processes underlying psychopathology.

In summary, NTRK2 modulates glutamate receptor function and synaptic plasticity in the amygdala, which is centrally implicated in emotional arousal in health and mental disease. Thus, we hypothesized that *NTRK2* genetic variants are associated with emotional arousal independently of disease status. Specifically, we quantified the arousal ratings of emotional (negative and positive) and neutral IAPS pictures in two independent samples of healthy young subjects (*n*_1_=1171, *n*_2_=707). Since genetic variants in genes encoding for neurotrophic receptors have been associated with white-matter measures,^44, 45^ we analyzed in a subsample of 342 subjects white-matter properties as assessed by diffusion tensor imaging (DTI) in relation to arousal ratings, as well as *NTRK2* genotype. In this study, we focused on (a) fractional anisotropy (FA), which is a measure of the directional dependence of diffusion^46^ and reflects fiber density, as well as coherence within a voxel,^47^ and (b) mean diffusivity (MD), which reflects the magnitude of water diffusion within a voxel and depends on the density of physical obstructions like membranes and the distribution of water molecules between different cellular compartments.^47, 48^

## Materials and methods

### Participants

In total, we analyzed data of *N*=1878 subjects from 2 independent samples, that is, a hypothesis-testing (Basel_1) and a hypothesis-confirming sample (Basel_2). Overall, 65% of the subjects were female and the mean age was 23±3 years (age-range 18–35; for information about each sample separately see Supplementary Table S1). Subjects were recruited from the area of Basel in Switzerland. Sampling strategy was to recruit large samples of healthy young adults, without further restrictions. Advertising was done mainly in the University of Basel. Subjects were free of any neurological or psychiatric illness, and did not take any medication at the time of the experiment. The ethics committee of the Cantons of Basel City and Basel Country approved the experiments. Written informed consent was obtained from all subjects prior to participation.

### Behavioral tasks

All subjects performed an identical picture task. This task consisted of the presentation of 24 IAPS pictures per valence group (negative, neutral and positive), as well as pictures from in-house standardized picture sets. Subjects rated the presented pictures according to valence (positive=1, neutral=0, negative=−1) and arousal (high=3, medium=2, low=1). For more detailed sample task and procedure description see Supplementary I paragraph *Procedure and task description*.

### Saliva DNA samples collection and isolation

Saliva samples were collected from all subjects at the time-point of the main investigation, using Oragene DNA Kit (DNA Genotek, Ottawa, ON, Canada). Saliva DNA was extracted from the Oragene DNA Kit using the standard precipitation protocol recommended by the producer.

### Array-based SNP genotyping

Samples were processed as described in the Genome-Wide Human SNP Nsp/Sty 6.0 User Guide (Affymetrix, Santa Clara, CA, USA). Briefly, genomic DNA concentration was determined by using a Nano-Drop ND-1000 and adjusted to 50 ng ml^−1^ in water; 250 ng of DNA was digested in parallel with 10 U of *Sty*I and *Nsp*I restriction enzymes (New England Biolabs, Ipswich, MA, USA) for 2 h at 37 °C. Enzyme-specific adaptor oligonucleotides were then ligated onto the digested ends with T4 DNA Ligase for 3 h at 16 °C. After adjustment to 100 ml with water, 10 ml of the diluted ligation reactions were subjected to PCR. Three PCR reactions of 100 ml were performed for Sty-digested products and four PCR reactions for Nsp. PCR was performed with Titanium Taq DNA Polymerase (Clontech, Mountain View, CA, USA) in the presence of 4.5 mm PCR primer 002 (Affymetrix), 350 mm each dNTP (Clontech), 1 m G-C Melt (Clontech) and 13 Titanium Taq PCR Buffer. Cycling parameters were as follows: initial denaturation at 94 °C for 3 min, amplification at 94 °C for 30 s, 60 °C for 45 s and extension at 68 °C for 15 s repeated a total of 30 times, final extension at 68 °C for 7 min. Reactions were then verified to migrate at an average size between 200–1100 bp using 2% TBE gel electrophoresis. PCR products were combined and purified with the Filter Bottom Plate (Seahorse Bioscience, North Billerica, MA, USA) using Agencourt Magnetic Beads (Beckman Coulter, Brea, CA, USA). Purified PCR products were quantified on a Zenith 200rt microplate reader (Anthos-Labtec, Salzburg, Austria). We obtained 4–5 mg ml^−1^ on average for each sample. From this stage on, the SNP Nsp/Sty 5.0/6.0 Assay Kit (Affymetrix) was used. Around 250 mg of purified PCR products were fragmented using 0.5 U of DNase I at 37 °C for 35 min. Fragmentation of the products to an average size <180 bp was verified using 4% TBE gel electrophoresis. After fragmentation, the DNA was end labeled with 105 U of terminal deoxynucleotidyl transferase at 37 °C for 4 h. The labeled DNA was then hybridized onto Genome-Wide Human SNP 6.0 Array at 50 °C for 18 h at 60 r.p.m. The hybridized array was washed, stained and scanned according to the manufacturer's (Affymetrix) instructions using Affymetrix GeneChip Command Console (AGCC, version 3.0.1.1214, Affymetrix). Generation of single nucleotide polymorphisms (SNP) calls and Array quality control (QC) were performed using the command line programs of the Affymetrix Power Tools package (version: apt-1-14.4.1, Affymetrix). According to the manufacturer's recommendation, contrast QC was chosen as QC metric, using the default value of ⩾0.4. All samples passing QC criteria were subsequently genotyped using the Birdseed (v2) algorithm (Broad Institute, Cambridge, MA, USA). Mean Call Rate for all samples averaged >98.4%. This value refers to per sample (that is, individual) call rate and ranged from 93 to 100%.

### SNP selection

Generation of SNP calls and array QC were performed using the Affymetrix Genotyping Console Software 3.0 (Affymetrix). Genotypic outliers were identified and removed using Bayesian clustering algorithm^49^ (for more details see Supplementary II paragraph *Sample QC with Bayesian clustering algorithm*).

SNPs on the *NTRK2* gene (50 kb upstream and 10 kb downstream, chr9:87,233,466-87,648,505) based on University of California Santa Cruz (UCSC, http://genome.ucsc.edu) annotation hg19 were used for analysis. For association testing, markers with genotype call rate <0.98, with minor allele frequency <0.01 and with Hardy–Weinberg equilibrium *P*<0.05 were excluded leading to a reduction from 135 markers to 85 markers to be analyzed.

### Genetic association analysis

Analyses of variance were calculated with the WG-Permer software (www.wg-permer.org). This software corrects nominal *P*-values for multiple testing on a permutation-based procedure (number of permutations set to 10 000) according to the study by Westfall and Young.^50^ Dominant and recessive models of inheritance were calculated.

Mean arousal ratings per valence category (positive, negative and neutral) of IAPS pictures served as quantitative phenotypes. Since sex- and age-related differences in emotional processing are observed,^51, 52^ we corrected for these variables by using the *z*-transformed residuals derived from a linear regression.

In the hypothesis-testing sample, behavioral data for the phenotypes of interest were available for 1552 subjects, of whom 1389 subjects had genetic data. After QC, we excluded 160 subjects (genetic background outliers: 154 subjects, gender inconsistencies: 6 subjects). In total 1171 subjects had a complete data set (behavioral and genetic data). The hypothesis-confirming sample comprised initially 1024 subjects with behavioral data on the phenotypes of interest. Of these 1024 subjects, 827 subjects had genetic data. After QC, we excluded 104 subjects (genetic background outliers: 97 subjects, gender inconsistencies: 7 subjects). In total, 707 subjects had a complete data set (behavioral and genetic data).

In addition to the candidate gene analysis, a genome-wide association study of emotional arousal was performed with the array data. PLINK^53^ was used for association testing under the dominant, recessive and additive genetic model. SNPs with a genotype call rate <0.99, a minor allele frequency <0.01 and with a Hardy–Weinberg equilibrium *P*<0.001 were excluded. Only Bonferroni-corrected *P*-values <5 × 10^−8^ were considered significant.

### DTI data acquisition and analysis

Diffusion volumes were acquired on a 3 T scanner using a single-shot echo-planar sequence, and consisted of 64 diffusion-weighted volumes (*b*=900 s.mm^−2^) and 1 unweighted volume (*b*=0 s.mm^−2^). Diffusion data were analyzed using FMRIB Software Library v4.1.7 (FSL, http://www.fmrib.ox.ac.uk/fsl). Maps of FA and MD were obtained from the diffusion tensor (DT) model for further analyses. Voxel-wise statistical analyses of FA and MD maps were carried out using the Tract-Based Spatial Statistics (TBSS) toolbox of FSL.^54^ Voxel-wise statistical analyses were run on the skeletonized FA and MD maps using permutation-based nonparametric inference within the framework of the general linear model^55^ as implemented in the randomise tool.^56^ Five thousand permutations were performed and results were considered significant for *P*<0.05, corrected for multiple comparisons across space using the 'two-dimensional' parameter settings with threshold-free cluster enhancement, which avoids using an arbitrary threshold for the initial cluster-formation.^57^ Display of the results was done using the tbss_fill command to ease visualization. The significant voxels were labeled according to the John Hopkins University white-matter tractography atlas available in FSL.^58, 59^ For more detailed information about data acquisition, processing and quantification of results see Supplementary III paragraph *Imaging data acquisition and analysis*. These data were only available in the hypothesis-confirming sample. Out of 707 subjects with complete data sets from the genetic association analysis, 342 subjects had complete DTI, mean arousal rating and genotype data.

### DTI analysis—genotype-independent association analyses between DTI measures and mean positive arousal rating

The linear associations between mean positive arousal ratings and FA, as well as MD were tested, including age and sex as covariates. We also performed analyses of first eigenvalue (L1) and radial diffusivity.

### DTI analysis—genotype-dependent differences in MD and FA

Further, we compared voxel-wise MD and FA values between genotype groups using a linear model, including age and sex as covariates.

### DTI analysis—genotype-dependent differences in tracts with a significant association between MD and mean positive arousal rating

In a linear association analysis, we tested the significance of the interaction term between mean positive arousal and genotype using the averaged MD value per subject from all the voxels showing a significant association between mean positive arousal ratings and MD as dependent variable.

## Results

### Genetic association analysis

**Figure 1:**
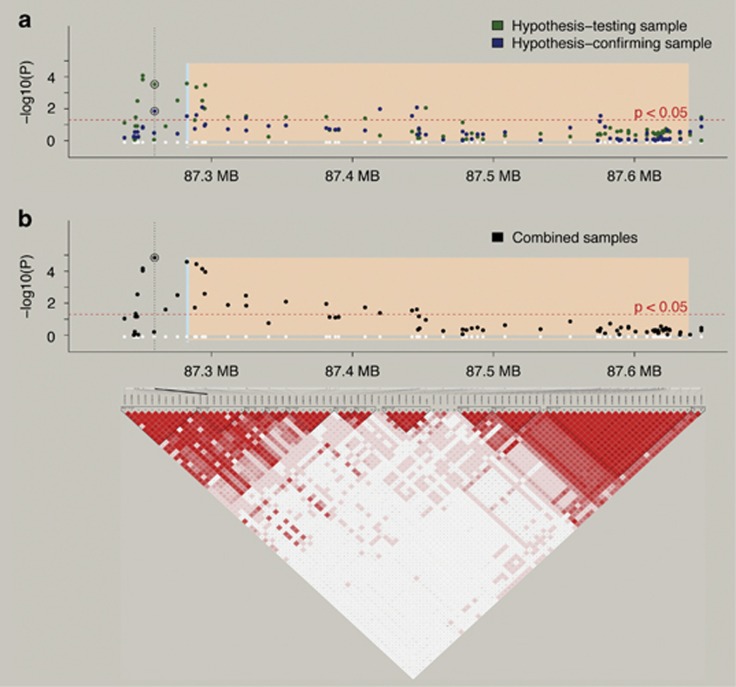
Association between 85 *NTRK2* gene SNPs (recessive model) and mean positive arousal ratings (**a**) for the hypothesis-testing and -confirming sample separately as well as (**b**) in the combined sample. In **a** and **b**, the significant SNP rs2579372 is marked by a circle and its position is highlighted by a dotted vertical line. The white dots indicate the physical location of the 85 SNPs. The gene- and promoter-region are depicted by the peach and turquoise color, respectively. On the *y*-axis log-transformed *P*-values are represented and the *x*-axis depicts the chromosomal location in mega base pairs. The red dashed line represents the uncorrected *P*-value level <0.05. The lower panel shows the linkage disequilibrium (LD) structure of the *NTRK2* locus in the hypothesis-testing sample. LD, linkage disequilibrium; SNP, single nucleotide polymorphism.

In the hypothesis-testing sample (*N*=1171), the genotype–phenotype association of four SNPs survived Westfall Young correction for multiple testing (number of tested SNPs: 85, number of phenotypes: 3). All SNPs showed a significant association with mean arousal ratings for positive pictures. The association between these SNPs and mean arousal ratings for negative or neutral pictures was not significant, neither at the corrected nor at the nominal significance level (for an overview see Supplementary Table S2). The association with mean arousal ratings for positive pictures was replicated for 1 of the 4 SNPs (rs2579372) in the hypothesis-confirming sample of 707 subjects (Table 1). The direction of effect and genetic model (recessive) were the same as in the hypothesis-testing sample (for an overview see Figure 1). Specifically, T-allele carriers of rs2579372 rated positive pictures as more arousing than non-T-allele carriers.

The additionally performed genome-wide analysis did not reveal any variant exceeding Bonferroni-corrected (*P*<5 × 10^−8^) significance.

### Genotype-independent association between DTI measures and mean positive arousal rating

**Figure 2:**
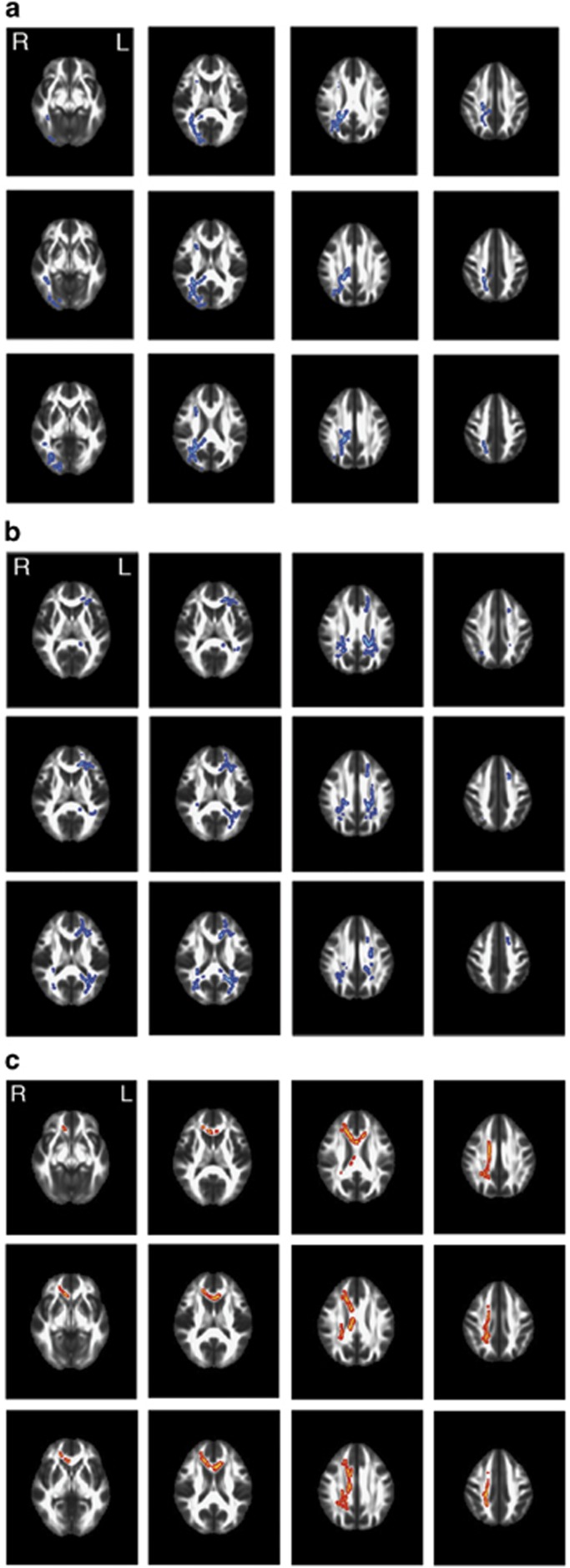
(**a**) Tracts with genotype-independent negative correlations between mean positive arousal ratings and MD (whole-brain corrected *P*_FWE_<0.05). (**b**) Tracts with significant (whole-brain corrected *P*_FWE_<0.05) genotype-dependent differences in MD. (**c**) Tracts with significant (whole-brain corrected *P*_FWE_<0.05) genotype-dependent differences in FA. FA, fractional anisotropy; L, left; MD, mean diffusivity; R, right.

In 342 subjects of the hypothesis-confirming sample, we observed a significant whole-brain-corrected (*P*_FWE_<0.05) negative correlation between mean positive arousal ratings and MD (that is, higher MD correlated with lower mean arousal ratings for positive pictures) in several tracts of the right hemisphere, among others in the cingulum, inferior and superior longitudinal fasciculus (see Figure 2a and Table 2a). There was no significant (whole-brain corrected *P*_FWE_<0.05) positive correlation between MD and mean positive arousal ratings. Neither a negative nor a positive significant (whole-brain corrected *P*_FWE_<0.05) correlation between FA and mean positive arousal ratings was detected. We observed a whole-brain corrected (*P*_FWE_<0.05) negative correlation between mean positive arousal ratings and L1 (that is, higher L1 correlated with lower mean arousal ratings for positive pictures) bilaterally in the superior longitudinal fasciculus and several tracts of the right hemisphere including the anterior thalamic radiation, cingulum, corticospinal tract, inferior fronto-occipital and longitudinal fasciculus, and uncinate, as well as forceps minor and major (see Supplementary Table S3). No whole-brain corrected (*P*_FWE_<0.05) positive association between L1 and mean positive arousal ratings was observed. The radial diffusivity analysis did not reveal any significant (whole-brain corrected *P*_FWE_<0.05) positive or negative associations with mean positive arousal.

### Genotype-dependent differences in MD and FA

**Figure 3:**
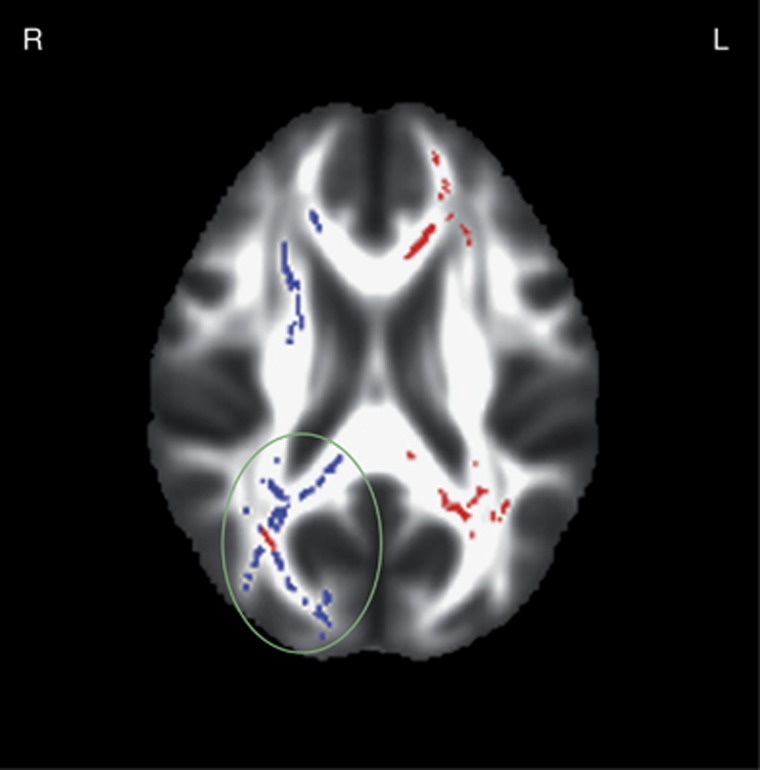
Overlap of significant (whole-brain corrected *P*_FWE_<0.05) genotype-independent negative correlations between MD and mean positive arousal ratings (in blue color) and significant (whole-brain corrected *P*_FWE_<0.05) genotype-dependent MD differences (in red color) exemplarily in the forceps major and inferior fronto-occipital fasciculus (green circle). For a brain-wide overview see Supplementary Figure S1 in the supplementary. MD, mean diffusivity.

We identified a number of tracts with whole-brain-corrected genotype-dependent differences in MD, with T-allele carriers (*n*=276) of SNP rs2579372 having higher MD than non-T-allele carriers (*n*=66). These differences were mainly located bilaterally in white-matter fiber bundles connecting structures of the frontal and parietal lobes (see Figure 2b and Table 2b). There were no tracts showing significantly (whole-brain corrected *P*_FWE_<0.05) lower MD in T-allele carriers. In six out of the nine tracts showing a significant association between mean arousal ratings of positive pictures and MD, we observed as well significant differences in MD between genotype groups (see Figure 3 and Table 2 tracts marked with superscript c) on a tract- but not voxel-level.

In addition, we observed a number of tracts with whole-brain corrected genotype-dependent differences in FA, with T-carriers (*n*=276) of SNP rs2579372 having lower FA than non-T-allele carriers (*n*=66). These differences were mainly located in the right hemisphere in white-matter fiber bundles connecting structures of the frontal and parietal lobes (see Figure 2c and Table 2c).

### Genotype-dependent differences in tracts with a significant association between MD and mean positive arousal rating

Due to the overlap between significant tracts for the association of MD and mean arousal ratings of positive pictures, as well as genotype-dependent MD differences, we finally tested only in these overlapping voxels for a phenotype (mean positive arousal) × genotype interaction. There was no significant whole-brain-corrected (*P*_FWE_<0.05) interaction.

## Discussion

We showed that a common SNP 5′ to *NTRK2* (rs2579372) is associated with emotional arousal in two independent samples of healthy young subjects. T-allele carriers rated positive pictures as more arousing compared with non-T-allele carriers. In a genotype-independent analysis, we observed a negative correlation between mean positive arousal ratings and MD, a measure of brain white-matter integrity. We also observed rs2579372 genotype-dependent regional differences in MD and FA, with T-allele carriers having higher MD, respectively, lower FA values than non-T-allele carriers. Interestingly, the tracts showing genotype-dependent MD differences largely overlapped with the tracts showing significant correlations between MD and emotional arousal. However, the *NTRK2* effects on emotional arousal and on MD were independent.

The genotype-dependent differences in emotional arousal ratings reported herein argue in favor of an involvement of *NTRK2* in emotional processing in healthy subjects. To our knowledge this is the first report on the relation between *NTRK2* and emotional processing in healthy subjects. So far several genetic associations between *NTRK2* and psychopathology have been reported in the literature.^31, 32, 33, 34, 35, 36, 37, 38, 39, 40, 60, 61, 62^ Given that dysregulation of emotional processes is a common characteristic of psychiatric disorders^1^ and that emotional arousal—specifically, the rating of emotionally arousing IAPS pictures—is reportedly altered in patients with psychiatric disorders,^4, 5, 6, 7, 8, 9^ we hypothesize that *NTRK2* variants might be related to psychopathology through their influence on emotional processing. However, no study has hitherto investigated the direct link between *NTRK2* and emotional dysregulation in psychopathology, and our results allow inferences solely in healthy populations. Nevertheless, the association between *NTRK2* and emotional processing observed herein provides further ground for the investigation of the role of *NTRK2* in emotional processes in patients with psychiatric disorders.

In our DTI analyses, we observed negative correlations between mean positive arousal ratings and a white-matter microstructure property (MD) in tracts that are mainly part of the limbic circuits (anterior thalamic radiation and cingulate gyrus) or form connections from other brain regions to these circuits (inferior fronto-occipital, inferior and superior longitudinal fasciculus).^63^ The limbic circuitry, along with its connections to other brain regions plays an essential role in emotion processing.^29^ The results of our DTI analysis are in line with this evidence. In addition, several studies report significant alterations in these tracts in patients with different psychiatric disorders like obsessive compulsive disorder, bipolar disorder, autism spectrum disorder, antisocial personality disorder and schizophrenia.^64, 65, 66, 67, 68^ Although the *NTRK2* effects on emotional arousal and on MD were independent, it is of interest to note that the genotype-dependent differences in MD reported herein were regionally overlapping with the results of the correlation between arousal ratings and MD, and that engaged areas are involved in emotional processing^29^ and psychiatric disorders.^64, 65, 66, 67, 68^ In addition, we identified several tracts showing genotype-dependent differences in FA values (albeit in the absence of significant whole-brain-corrected correlations between FA and emotional arousal). Interestingly, these tracts overlapped substantially with the tracts showing genotype-dependent MD differences. Of note, Braskie *et al.*^44, 45^ reported significant *NTRK1* and *NTRK3* genotype-dependent differences in FA values in partially the same tracts, in which we identified *NTRK2* genotype-dependent MD and FA differences. In our sample, the correlation between FA and MD values was highly significant (whole-brain average *r*_(FA,MD)_=−0.75, *P*=9.9e–128).

In addition to MD and FA, we also analyzed L1 and radial diffusivity values and observed significant correlations between L1 and mean positive arousal. L1 represents the largest eigenvalue of the fitted tensor or parallel diffusivity. We would like to stress that it is rather difficult to link this association to a specific axon-related characteristic (that is, axon diameter, axon count or axon density), given that the population under study consists uniquely of healthy young participants.^69^ Furthermore, because of the limitation of the tensor model in resolving regions of complex fiber architecture (for example, crossing fibers),^70^ the interpretation of DTI results to specific tracts is inherently limited. More advanced acquisition methods, such as high-angular-resolution diffusion imaging or diffusion spectrum imaging, might help disentangling this issue.^71^ These approaches are, however, usually more time-consuming and limit the investigation of large cohorts often needed in imaging genetics. Despite these methodological limitations, our results support an influence of neurotrophic receptor gene variants on white-matter microstructure.

In summary, our results suggest the involvement of a *NTRK2* tag SNP in emotional processing and—independently—in brain white-matter integrity in healthy young subjects. Both the correlation of MD values with emotional arousal and the *NTRK2* genotype-dependent differences in MD and FA values were located in brain regions known to be important for emotion processing in health and disease. These results may provide guidance for future studies on the role of *NTRK2* in the mechanisms of emotion dysregulation.

## Figures and Tables

**Table 1:** Genetic association of rs2579372 in the hypothesis-testing and -confirming samples

					*Homozygous minor allele carriers*			*Heterozygous and major allele carriers*			
*SNP*	*Model*	P-*value nominal*	*WY (SNP+PT)*	*Minor/major allele*	N	*Mean*	*s.d.*	N	*Mean*	*s.d.*	*Total* N
*Hypothesis-testing sample*											
rs2579372	Carrier T	0.0003	0.0478	C/T	268	−0.18	0.95	893	0.06	1.00	1161

					*Homozygous minor allele carriers*			*Heterozygous and major allele carriers*			
*SNP*	*Model*	P-*value nominal*	*WY (SNP)*	*Minor/major allele*	N	*Mean*	*s.d.*	N	*Mean*	*s.d.*	*Total* N
*Hypothesis-confirming sample*											
rs2579372	Carrier T	0.0141	0.0356	C/T	134	−0.20	1.01	573	0.03	0.98	707

Abbreviations: *N*, number of subjects; PT, phenotype; SNP, single nucleotide polymorphism; WY, permutation-based correction for multiple testing according to Westfall Young for SNP and/or PT.

**Table 2:** Tracts showing significant (whole-brain corrected *P*_FWE_<0.05) (A) phenotype- or (B) genotype-dependent association with MD and (C) FA

*Tracts*	*Hemisphere*	*Min. corrected*	*Min. uncorrected*
		P-*value*^a^	P-*value*^b^
*(A) Tracts showing a significant (whole-brain corrected* P_FWE_*<0.05) association between mean positive arousal ratings and MD*			
Anterior thalamic radiation	R	0.0444	0.0018
Cingulum (cingulate gyrus)	R	0.0348	0.0036
Corticospinal tract^c^	R	0.0400	0.0036
Forceps major^c^		0.0348	0.0016
Forceps minor^c^		0.0460	0.0022
Inferior fronto-occipital fasciculus^c^	R	0.0324	0.0026
Inferior longitudinal fasciculus	R	0.0356	0.0030
Superior longitudinal fasciculus (temporal part)^c^	R	0.0334	0.0044
Superior longitudinal fasciculus^c^	R	0.0334	0.0044
			
*(B) Tracts showing significant (whole-brain corrected* P_FWE_*<0.05) genotype-dependent differences in MD*			
Anterior thalamic radiation	L	0.0366	0.0026
Cingulum (cingulate gyrus)	L	0.0436	0.0036
Corticospinal tract^c^	L	0.0366	0.0054
	R	0.0490	0.0048
Forceps major^c^		0.0440	0.0024
Forceps minor^c^		0.0454	0.0030
Inferior fronto-occipital fasciculus^c^	L	0.0456	0.0018
	R	0.0482	0.0060
Inferior longitudinal fasciculus	L	0.0454	0.0016
Superior longitudinal fasciculus (temporal part)^c^	L	0.0456	0.0046
	R	0.0494	0.0066
Superior longitudinal fasciculus^c^	L	0.0454	0.0026
	R	0.0494	0.0038
Uncinate fasciculus	L	0.0460	0.0042
			
*(C) Tracts showing significant (whole-brain-corrected* P_FWE_*<0.05) genotype-dependent differences in FA*			
Anterior thalamic radiation	R	0.0338	0.0020
Cingulum (cingulate gyrus)	L	0.0336	0.0044
	R	0.0352	0.0026
Corticospinal tract	R	0.0244	0.0010
Forceps minor		0.0322	0.0020
Inferior fronto-occipital fasciculus	R	0.0260	0.0034
Superior longitudinal fasciculus (temporal part)	R	0.0338	0.0008
Superior longitudinal fasciculus	R	0.0250	0.0010
Uncinate fasciculus	R	0.0468	0.0022

Abbreviations: FA, fractional anisotropy; L, left hemisphere; MD, mean diffusivity; R, right hemisphere.

a Represents the smallest whole-brain corrected *P*-value in the tract.

b Represents the smallest uncorrected *P*-value in the tract.

c Tracts showing both significant (whole-brain corrected *P*_FWE_<0.05) associations between mean positive arousal ratings and MD, as well as a significant (whole-brain corrected *P*_FWE_<0.05) genotype-dependent MD differences.
