# Supplementary figures and images for: A common NTRK2 variant is associated with emotional arousal and brain white-matter integrity in healthy young subjects
Source: Transl Psychiatry. 2016 Mar 15;6(3):e758–. doi: 10.1038/tp.2016.20 (PMC4872446; doi:10.1038/tp.2016.20)

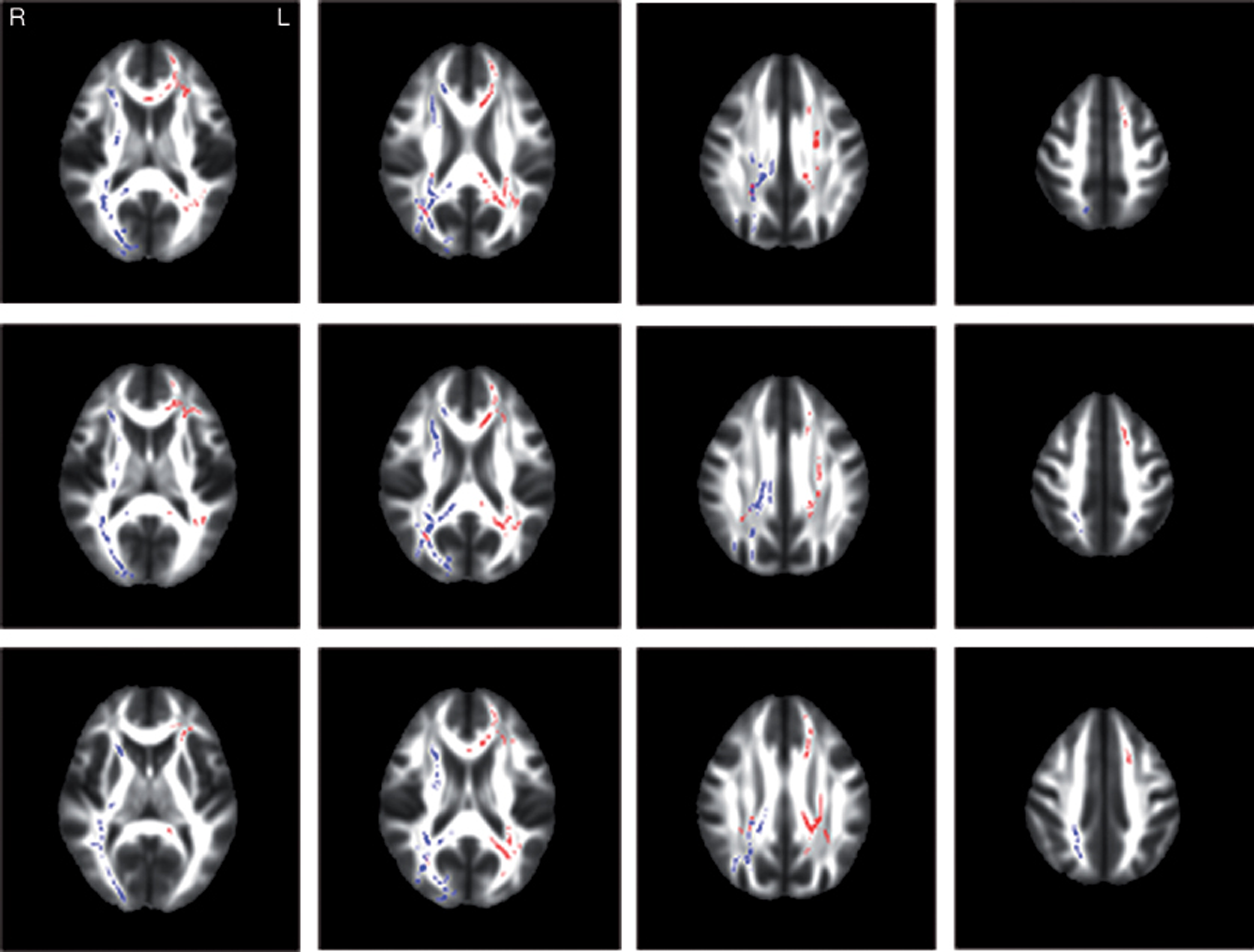

Supplement: Supplementary Figure 1 [file tp201620x1.tif]
