# Supplementary material for: A common NTRK2 variant is associated with emotional arousal and brain white-matter integrity in healthy young subjects
Source: Transl Psychiatry. 2016 Mar 15;6(3):e758–. doi: 10.1038/tp.2016.20 (PMC4872446; doi:10.1038/tp.2016.20)
Supplement: Supplementary Information [file tp201620x2.doc]

**Supplemental information**

**Table S1**: Overview subjects per sample

^1^SD=standard deviation, ^2^N=number of subjects

| **Variables** | **Samples** | |
| --- | --- | --- |
|  | **hypothesis-testing**  **sample** | **hypothesis-confirming**  **sample** |
|  |  |  |
| **Sex (women/men)** | 791/380 | 421/286 |
|  |  |  |
| **Age (mean ± SD^1^)** | 23.01 ± 3.56 | 22.89 ± 3.24 |
|  |  |  |
| **Total N^2^** | 1171 | 707 |

*Supplemental I: Procedure and task description*

The experiment of both samples, the hypothesis-testing and -confirming sample, was conducted in Basel. The task used here for analyses was in both samples identical. In the hypothesis-testing sample subjects performed the same task two times. To ensure comparability between the samples we only considered the first performance on the task in that sample. The experimental procedures of the two samples were different.

*Picture task*

We used an event-related design consisting of 100 trials (including 2 primacy and 2 recency trials, 24 scrambled pictures, and 72 pictures). Stimuli consisted of 72 pictures that were selected from the IAPS (IAPS; ^1^) as well as from in-house standardized picture sets that allowed us to equate the pictures for visual complexity and content (e.g. human presence; in total only 8 pictures). On the basis of normative valence scores (from 1 to 9), pictures were assigned to emotionally negative (2.3 ± 0.6), emotionally positive (7.6 ± 0.4), and emotionally neutral (5.0 ± 0.3) conditions, resulting in 24 pictures for each emotional valence. Four additional IAPS pictures showing neutral objects were used to control for primacy and recency effects in memory. Two of these pictures were presented in the beginning and two at the end of the picture task. They were not included in the analysis. In addition to the IAPS pictures, 24 scrambled pictures were used. The scrambled pictures consisted of a colored background, containing the colour information of all pictures used in the experiment (except primacy and recency pictures), overlaid with a crystal and distortion filter (Adobe Photoshop CS3). In the foreground, a mostly transparent geometrical object (rectangle or ellipse of different sizes and orientations) was shown.

The pictures were presented for 2.5 seconds in a quasi-randomized order so that at maximum four pictures of the same category occurred consecutively. A fixation-cross appeared on the screen for 500 ms before each picture presentation. Trials were separated by a variable intertrial period (period between appearance of a picture and the next fixation cross) of 9-12 seconds (jitter). During the intertrial period, participants subjectively rated the picture showing scenes according to valence (positive=1, neutral=2, negative=3; recoded afterwards to positive=1, neutral=0, negative=-1) and arousal (high=1, medium=2, low=3; recoded afterwards to high=3, medium=2, low=1) on a three-point scale (Self Assessment Manikin, SAM) by pressing a button with a finger of their dominant hand. For scrambled pictures, participants rated form (vertical=1, symmetric=2, horizontal=3) and size (small=1, medium=2, large=3) of the geometrical object in the foreground. The total duration of the picture task was 20 minutes. Participants were instructed and trained on the picture task before the task performance. Training consisted of presentation and rating of five pictures including scenes and scrambled pictures, which were not used during the main task.

*Hypothesis-testing sample - Procedure*

The experiments were conducted on three dates, where two were on two consecutive days. Subjects received general information about the study and gave their written informed consent on the first date. On the second date participants received instructions and were trained on the picture task, amongst others. After training, participants performed on the picture task for 20 minutes. On the third date, participants completed again the picture task (20 minutes) with a new set of emotional and neutral pictures. On the last two dates, participants filled in health and psychological questionnaires and were debriefed at the end of the experiment. The total length of the experiment procedure was approximately 6 to 6.5 hours. Participants received 25.- CHF/h for participation.

*Hypothesis-confirming sample - Procedure*

After receiving general information about the study and giving their written informed consent, participants were instructed and then trained on the picture task they later performed. After having performed on some tasks, subjects went in the MR-scanner for the diffusion tensor imaging (DTI) measurements. Scans were acquired for 10 minutes. Finally, participants filled in health and psychological questionnaires and were debriefed. The total length of the experiment procedure was approximately 3 to 4.5 hours. Participants received 25.- CHF/h for participation.

*Supplemental II: Sample quality control with bayesian clustering algorithm*

Within each center Bayesian Clustering Algorithm ^2^ was applied on genome-wide summary statistics to identify and exclude atypical samples. Considering a combination of two summary statistics, the algorithm infers each sample's posterior probability to belong to the outliers' class. A first outlier assessment was based on genome-wide call rate and heterozygosity rates, for which extreme values may be indicative of a genotyping bias. The second assessment aimed at identifying subjects with unusual ancestry according to the majority of the samples. This was done by projecting the samples genotypic data on the two first PCA components inferred from HapMap data using YRI, CEU and CHB-JPT populations. Samples were also checked for consistency between genetically inferred and self-reported gender.

*Supplemental III: Imaging data acquisition and analysis*

Diffusion volumes were acquired using a single-shot echo-planar sequence, and consisted of 64 diffusion-weighted volumes (b=900 s/mm2) and one unweighted volume (b=0 s/mm2). Acquisition parameters were as following: TR=9000 ms, TE=82 ms, FOV=320 mm, GRAPPA R=2.0, voxel size 2.5x2.5x2.5 mm3. In total, 346 subjects of the imaging sample had diffusion-weighted data.

Diffusion data were analyzed using FSL v5.0.2 (<http://www.fmrib.ox.ac.uk/fsl>). DWI volumes were first visually inspected to detect any corrupted directions, e.g. due to motion ^3^. In subjects where more than 1 direction was affected, those corrupted volumes were removed before proceeding. Two subjects for which more than 5 directions were affected were excluded from the analysis. Diffusion volumes were first coregistered to the reference unweighted volume (b=0 s/mm2) using an affine transformation for the correction of head motion and eddy current induced image distortion. After removal of non-brain tissue, a diffusion tensor (DT) model was fitted on a voxel-by-voxel basis. Maps of fractional anisotropy (FA) and mean diffusivity (MD) were obtained from the DT model for further analyses.

Voxelwise statistical analysis of FA and MD maps was carried out using the Tract-Based Spatial Statistics (TBSS) toolbox of FSL ^4^. FA volumes were first non-linearly warped to FSL’s high-resolution FA template (FMRIB58_FA) in the MNI152 space using the non-linear registration tool FNIRT. Next, a mean FA volume was computed and thinned to generate a mean FA skeleton, representing the center of white matter bundles common to all subjects. The skeleton was thresholded at FA>0.2 in order to reduce partial volume effects between white matter and other tissue classes. Finally, perpendicular projection of local maximal FA values onto the skeleton was done for each subject, accounting for residual variations in alignment. The computed non-linear warp and skeleton projection were also applied to the MD volumes, to create skeletonized MD maps.

*Quantification of the results*

Labeling of the significant voxels was done according to the John Hopkins University (JHU) white-matter tractography atlas ^5,6^ provided with FSL. For each of the twenty major white matter tracts included in the atlas, a tract of interest was created by applying a threshold of 10% to the probability map.

**Table S2**: Associations with mean negative and neutral arousal ratings of SNPs showing a significant association with mean positive arousal rating in the hypothesis-testing sample.

| **SNP** | **mean arousal rating** | **Model** | **p value nominal** | **WY^1^ (SNP+PT)** | **major/minor allele** | **homozygous**  **major allele carriers** | | | **heterozygous carriers** | | | **homozygous**  **minor allele carriers** | | |
| --- | --- | --- | --- | --- | --- | --- | --- | --- | --- | --- | --- | --- | --- | --- |
|  |  |  |  |  |  | N^2^ | MEAN | SD^3^ | N^2^ | MEAN | SD^3^ | N^2^ | MEAN | SD^3^ |
| **rs1212171** | positive | Carrier-A | 0.0003 | 0.045 | A/G | 356 | 0.02 | 1.05 | 554 | 0.09 | 0.95 | 261 | -0.19 | 0.95 |
|  | negative |  | 0.1080 | 1.000 |  |  | 0.02 | 1.03 |  | 0.06 | 0.95 |  | -0.07 | 0.98 |
|  | neutral |  | 0.2550 | 1.000 |  |  | -0.01 | 1.00 |  | 0.02 | 1.01 |  | -0.07 | 0.98 |
| **rs2579372** | positive | Carrier-T | 0.0003 | 0.048 | T/C | 348 | 0.03 | 1.05 | 545 | 0.09 | 0.95 | 268 | -0.18 | 0.95 |
|  | negative |  | 0.0678 | 0.999 |  |  | 0.01 | 1.02 |  | 0.07 | 0.95 |  | -0.07 | 0.98 |
|  | neutral |  | 0.2210 | 1.000 |  |  | -0.02 | 0.99 |  | 0.04 | 1.02 |  | -0.07 | 0.98 |
| **rs985542** | positive | Carrier-G | 0.0001 | 0.015 | G/C | 330 | -0.01 | 1.05 | 568 | 0.11 | 0.96 | 270 | -0.2 | 0.94 |
|  | negative |  | 0.1140 | 1.000 |  |  | 0.02 | 1.04 |  | 0.06 | 0.95 |  | -0.06 | 0.96 |
|  | neutral |  | 0.2550 | 1.000 |  |  | -0.03 | 1.00 |  | 0.03 | 1.02 |  | -0.07 | 0.98 |
| **rs985543** | positive | Carrier-T | 0.0002 | 0.025 | T/C | 332 | -0.0002 | 1.04 | 569 | 0.11 | 0.97 | 270 | -0.19 | 0.93 |
|  | negative |  | 0.1590 | 1.000 |  |  | 0.02 | 1.04 |  | 0.05 | 0.95 |  | -0.05 | 0.96 |
|  | neutral |  | 0.3190 | 1.000 |  |  | -0.03 | 1 |  | 0.03 | 1.02 |  | -0.06 | 0.98 |

^1^ permutation based correction for multiple testing according to Westfall Young for single nucleotide polymorphism (SNP) and phenotype (PT)

^2^ number of subjects

^3^ standard deviation

**Table S3**: Tracts showing significant (whole-brain corrected p_FWE_ < 0.05) between L1 and mean positive arousal.

| **Tracts** | **Hemisphere^1^** | **min. corrected**  ***p* – value^2^** | **min. uncorrected**  ***p* – value^3^** |
| --- | --- | --- | --- |
| Anterior thalamic radiation | R | 0.0288 | 0.0004 |
| Cingulum (cingulate_gyrus) | R | 0.0234 | 0.0006 |
| Corticospinal tract | R | 0.0240 | 0.0026 |
| Forceps major |  | 0.0384 | 0.0006 |
| Forceps minor |  | 0.0306 | 0.0006 |
| Inferior fronto-occipital fasciculus | R | 0.0252 | 0.0004 |
| Inferior longitudinal fasciculus | R | 0.0252 | 0.0004 |
| Superior longitudinal fasciculus | L | 0.0406 | 0.0008 |
|  | R | 0.0266 | 0.0006 |
| Superior longitudinal fasciculus (temporal_part) | L | 0.0406 | 0.0008 |
|  | R | 0.0266 | 0.0006 |
| Uncinate fasciculus | R | 0.0288 | 0.0008 |
|  |  |  |  |

^1^L = left hemisphere, R = right hemisphere

^2^represents the smallest whole-brain corrected *p* – value in the tract

^3^represents the smallest uncorrected *p* – value in the tract

**Figure S1**: Overlap of significant (whole-brain corrected, *p* < 0.05) genotype-independent negative correlations between MD values and mean positive arousal ratings (blue) and significant (whole-brain corrected, *p* < 0.05) genotype-dependent MD differences (red).

*References*

1 Lang PJ, Öhmann A, Vaitl D. The International Affective Picture System (slides). Gainesville, FL, 1988.

2 Bellenguez C, Strange A, Freeman C, Donnelly P, Spencer CCA. A robust clustering algorithm for identifying problematic samples in genome-wide association studies. *Bioinformatics* 2012; **28**: 134–135.

3 Sharman MA, Cohen-Adad J, Descoteaux M, Messé A, Benali H, Lehericy S. Impact of outliers on diffusion tensor and Q-ball imaging: clinical implications and correction strategies. *J Magn Reson Imaging* 2011; **33**: 1491–1502.

4 Smith SM, Jenkinson M, Johansen-Berg H, Rueckert D, Nichols TE, Mackay CE *et al.* Tract-based spatial statistics: voxelwise analysis of multi-subject diffusion data. *Neuroimage* 2006; **31**: 1487–1505.

5 Wakana S, Jiang H, Nagae-Poetscher LM, van Zijl PCM, Mori S. Fiber tract-based atlas of human white matter anatomy. *Radiology* 2004; **230**: 77–87.

6 Hua K, Zhang J, Wakana S, Jiang H, Li X, Reich DS *et al.* Tract probability maps in stereotaxic spaces: analyses of white matter anatomy and tract-specific quantification. *Neuroimage* 2008; **39**: 336–347.
